# Diagnosing the first patient: Integrating histopathology into an undergraduate gross anatomy course

**DOI:** 10.1002/ase.70042

**Published:** 2025-05-13

**Authors:** Kyle A. Robertson, Megan Kruskie, Jessica N. Byram

**Affiliations:** ^1^ Department of Anatomy, Cell Biology, & Physiology Indiana University School of Medicine West Lafayette Indiana USA; ^2^ Department of Anatomy, Cell Biology, & Physiology Indiana University School of Medicine Indianapolis Indiana USA

**Keywords:** histopathology, humanism, pathology, undergraduate gross anatomy

## Abstract

The incorporation of humanism through exploration of pathology in gross anatomy allows students to develop a deeper appreciation of the pathological basis of disease and to explore the impacts of pathology on donors' lives through clinicopathologic correlation of their findings. The purpose of this short communication was to describe and evaluate a pilot intervention that integrated histopathology into an existing humanism thread, First Patient Project (FPP), in an undergraduate, dissection‐based gross anatomy course. Five reflections were collected from each student (*n* = 18 students, 90 reflections) and a post‐course questionnaire collected data on student perceptions of the FPP and integrations of histopathology. Content analysis was used to analyze reflection and survey free response data, and descriptive statistics were performed on Likert‐style items using Excel. The questionnaire was completed by six students (33%) and five themes comprise the perceived impacts of the integration of histopathology into the FPP: pathology deepens anatomy knowledge, promotes career exploration, novice medical professionals, reflections of donor pathology and lifestyle, and general feedback on histopathology integration. Student reflections demonstrated that the histopathology component of the FPP improved clinical understanding of pathology, helped facilitate feelings of belonging in the medical profession, and allowed them to reflect on their own humanity as well as that of the donors. This ultimately demonstrated that histology and particular histopathology from anatomic donors were feasible and provided an avenue for curricular integration and adding another layer of appreciation of the donors' clinical history.

## INTRODUCTION

Anatomy educators are increasingly recognizing that the anatomical learning environment is a space where integrated learning can occur and other technical (e.g., diagnostic) and nontechnical, discipline‐independent skills (NTDIS; e.g., humanism) can be learned. The gross anatomy dissection laboratory, specifically, has been proposed as an ideal environment to both model and assess professionalism and to begin development of a medical professional identity.^1^, ^2^, ^3^ If provided the space and initiative to reflect upon the dissection experience, the body donor enables students to appreciate the humanism and uniqueness of the human body, along with the exploration of death irrespective of the underlying pathology.^3^, ^4^ This enables students to incorporate their lived experience and their nascent theoretical understanding of disease through active exploration of donor clinical history. As such, integrating pathology into gross anatomy dissection both formally and informally has been proposed as a holistic approach to study anatomy and disease processes.^3^, ^5^ In medical education, gross pathology and histopathology have been successfully integrated into gross anatomy courses at several institutions via collection of abnormal donor tissues for submission of a pathology report or presentation,^5^, ^6^, ^7^, ^8^, ^9^, ^10^, ^11^ and through integration of pathologists into their courses to assist students in exploring the cause of death,^12^ thus meeting the goal of integrating basic science and clinical education.

Regardless of the efforts of vertical integration, anatomy educators must ensure students have the requisite anatomy knowledge to move on to their next phase of training or practice. However, as professional schools reduce curricular time in anatomy,^13^ it calls into question how to deliver a values‐based anatomy experience without overburdening the compact curriculum. Furthermore, while medical students are expected to understand pathological states of disease, stand‐alone pathology courses are increasingly rare in current foundational science curricula^14^ while basic science and clinical education are becoming increasingly integrated.^15^ As such, one viable option for the formal introduction of humanistic values and exploration of pathology may be in anatomy education prior to entering professional school. Therefore, we decided to incorporate histopathology into an undergraduate, dissection‐based gross anatomy course.

Students enrolled in Cadaveric Human Anatomy (BIOL‐N 461) at Indiana University, Indianapolis, take part in a values‐based gross anatomy course that integrates dissection of whole‐body donors with written reflections and explorations of gross pathology within the First Patient Project (FFP^16^). While the goal of the FPP is to present pathology findings in their donors and correlate them with impacts on quality of life, previous iterations of the course had no method to confirm pathology. Attainment of a curriculum enhancement grant led to the incorporation of histopathology with their donors' history and gross pathology which allowed students to incorporate the cellular basis of disease to assist in telling their donors' story. As such, the purpose of this short communication is to present a multi‐method pilot evaluation of the impacts of integrating histopathology into the existing FPP by exploring the impacts of histopathology on the formation of diagnostic skills, understanding of anatomy and pathology, and humanizing the anatomical donor. Adding this histopathology content is expected to promote humanistic engagement with donors and improve their perceptions of their anatomical understanding and knowledge of the pathologic basis of disease.

### Theoretical framework and researcher positionality

This work is guided by a social constructivist paradigm which aligns with the concept of situated learning and legitimate peripheral participation as a source of professional socialization.^17^ We ascribe to a constructivist paradigm that acknowledges multiple, socially constructed realities and the subjectivity of constructed knowledge. Furthermore, we contend that no research is free from researcher influence, and we acknowledge our perspectives and backgrounds have influenced the design and analysis of this study. To be reflexive, we report all authors to be anatomy educators who approach anatomy education from a humanistic lens. Further, we view a humanistic lens to anatomy education as moving away from the anatomical donor as “a previously neglected, sidelined and objectivized subject within medical education” and moving toward reflection, connection, respect and the “enculturation into medical professionalism” with the anatomical donor the vehicle to assist in accomplishing these goals.^18^ ^(pXI)^.

## CURRICULAR INTERVENTION AND ASSESSMENT

### Course description

This study took place in the context of BIOL‐N 461, an advanced‐level undergraduate course for students interested in health professional programs (e.g., physical therapy, physician assistant, medicine, etc.). Specifically, for this cohort, there were five donors with 3–4 students per donor, with students assigned to their donor for the duration of the course. Students perform a complete dissection of the donor encompassing 26 total dissections. Students have completed a prerequisite anatomy course that utilizes anatomical models, vertebrate organ specimens, and optic microscopy. Guidance for dissections is based on preparatory readings of the textbook and customized dissector, live and prerecorded prosection demonstrations, and guidance from instructors and teaching assistants. Investigations of histopathology were integrated into the existing structure of the First Patient Project (FPP) which is a formal curricular component aimed at promoting humanization of the donor and consisted of several elements including: a formal presentation about whole body donation, focus on pathology through submission of Patient Reports, promotion of teamwork, reflection, and role modeling of professional behaviors. Additional details about the overall course structure can be found in^19^ and a description and outcomes of the FPP can be found in Robertson et al.^16^

### Project design

Each team was allotted 10 tissue specimens for histopathology processing. Course faculty assisted the students in identifying organs or tissues if those tissues had what appeared to be an abnormal or pathological appearance. Course faculty assisted the students in deciding which specimens to collect from their donor to ensure they were judicious about how they used their 10‐specimen allotment and ultimately assisted the students in the collection of specimens for processing. Each donor has a stated cause of death that helped to guide students and faculty to where specimens may be collected. For example, several of the donors were stated to have lung cancer of various subtypes, which provided students with the opportunity to visualize both the gross (macroscopic) as well as the microscopic morphology of malignancy.

The lead author (KAR) is a trained pathologist's assistant and demonstrated to students and faculty the best practices for collecting specimens for processing to ensure there was enough normal tissue adjacent to the pathological tissue to assist in interpretation and they were of appropriate thickness for processing (e.g., the size of a nickel). Furthermore, this brief introduction to tissue collection ensured that the selection of tissues was a minor addition of time as the collection occurred concurrently to the observations and discussions of gross pathology. Furthermore, with each group acquiring a limited number of tissues, the additional time required to integrate histopathology collection was minimal.

Tissue samples were transported to the Histology Core at the end of each unit (approximately once per month). Specimens were from previously embalmed specimens, so they were placed in 70% alcohol after collection. The Histology Core continued the dehydration process, embedded the tissue samples in paraffin wax, sectioned them using a microtome, placed the specimens on glass slides, and stained the specimens using a traditional histological stain (hematoxylin and eosin; H&E). Once glass slides were received, the specimens were transported to a pathology group to be digitized using a Leica Aperio Slide Scanner, which allows viewing on a virtual (i.e., computer‐based) histology platform. Following digitization, the slides were uploaded to a shared folder for each dissection team so they could be accessed at any time.

Students met with a faculty familiar with histopathology morphology (KAR) once per month as digital slides were received to discuss their specimens, interpretation, and discuss common features of pathologic change in disease at the cellular level. Following the meetings, the students were advised to further explore the pathology and report their findings within their First Patient Reports at the end of each unit. All students have access to medical resources through the medical library, including recommended resources such as pathology textbooks and atlases, UpToDate, AccessMedicine, and Clinical Key to aid in student understanding of pathophysiological processes and identification. At the end of each of the four units, students collectively constructed a patient report that detailed four components from the unit dissections: (1) Evidence of Lifestyle, (2) Pathology, (3) Impacts of Pathology on Quality of Life, and (4) Anatomic Variants. At the end of the course, students delivered a group presentation of their patients and their findings to the whole class. Students were encouraged to present their images and discussions of their histopathology findings during the FPP.

### Data collection

Data for this intervention were collected through student reflections and responses to an anonymous post‐course survey about the FPP. A total of five reflections from each student were included in the analysis and included the “First day” reflection and four‐unit reflections. The post‐course survey was administered through the Qualtrics XM online platform (Qualtrics, Provo, UT). An anonymous link was sent to the students through the course learning management system. Robertson et al.^16^ report on student perceptions of the FPP using a similar post‐course survey. However, one additional Likert item was added to the previous 10‐item style, and two additional free responses were added asking students to explore their experiences collecting, analyzing, and interpreting histopathology samples and diagnosing their first patient.

### Data analysis

Reflections and survey free responses were analyzed using inductive content analysis^20^ and sought to answer the following research question: How does the integration of histopathology into the FPP impact perceived development of diagnostic skills, understanding of anatomy and pathology, and humanization of the anatomical donor? One researcher (J.N.B.) developed a codebook by taking marginal notes and independently coding each reflection and survey free response. Codes were organized into a final codebook that was applied to the entire sample using Dedoose (SocioCultural Research Consultants, Manhattan Beach, CA). Finally, the researchers met to organize the codes into categories and interpret the results. Descriptive statistics were performed on Likert‐style items using Excel (Microsoft 2021). This study was granted exempt status by the Institutional Review Board at Indiana University (IRB # 10962).

Several trustworthiness measures were undertaken to ensure the credibility and rigor of this research. Each of the authors has formal training and experience in qualitative research. Authors were reflexive during the design, data collection, and data analysis phases of the project. Finally, the researchers met frequently during the project to discuss initial impressions of collected data and interpretations of the findings.

## OUTCOMES

**FIGURE 1:**
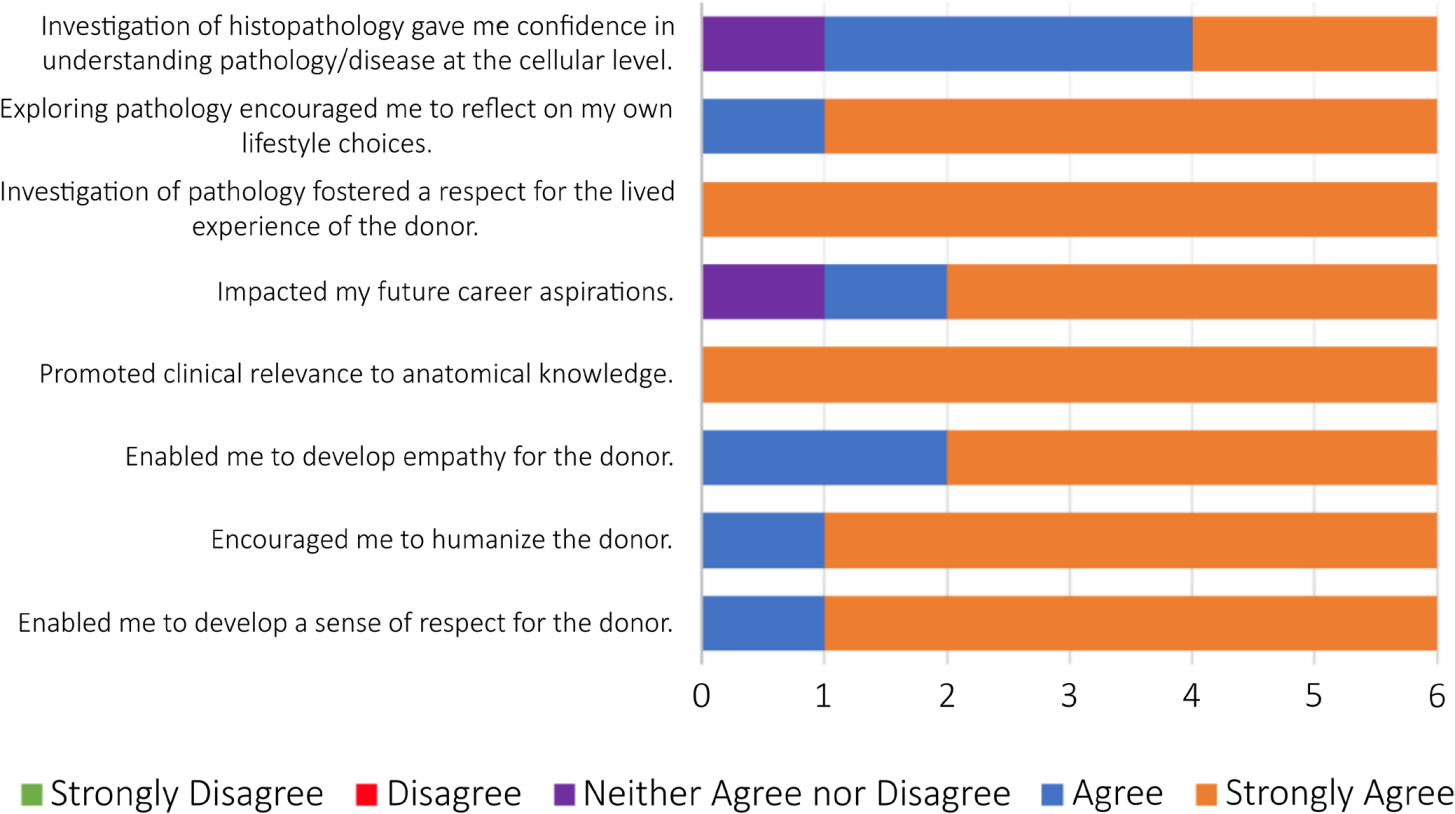
Select results from end of course survey of the FPP (*n* = 6).

The spring of 2024 cohort contained 18 students. As such, 90 reflections were analyzed, and students described impacts of histopathology incorporation into the FPP in each of the four unit reflections (*n* = 72). A total of six students (33%) completed the post‐course survey that provided Likert‐style and free responses regarding their perceptions of the FPP. Select items can be found in Figure 1 and demonstrate overall favorable perceptions of the FPP with histopathology, particularly in the areas of fostering respect for the donor and promoting clinical relevance. Furthermore, most students agreed that histopathology gave them the confidence to understand pathology and disease at the cellular level.

Five categories were identified to comprise the perceived impacts of the integration of histopathology into the FPP on the development of diagnostic skills, understanding of anatomy and pathology, and humanization of the anatomical donor in undergraduate students (Table 1).

**TABLE 1:** Categories, descriptions, and exemplary quotes on the impacts of integration of histopathology into the FPP (obtained from 72 student reflections and survey free responses).

Category	Description	Exemplary quote
Pathology deepens anatomy knowledge	Exploring diseases and their impacts on systems helped in understanding anatomy concepts	“It was interesting to see the interplay between diseased systems that caused further disease in other systems or structures. It has allowed for deeper appreciation of anatomy.” (Survey)
Promotes career exploration	Exploring diseases in organs created an interest in future medical specialties	“Being walked through the pathology of the tumor and getting to examine it myself, has helped me to realize that, while it is a devastating disease, cancer is fascinating to study and I could see myself working in a lab or becoming an oncologist.” (Unit 3 reflection)
Novice medical professional	Focusing on pathology as an undergraduate resulted in students feeling like they were becoming medical professionals	“Studying each unit and taking the time to understand the variants, evidence of lifestyle, pathology, and impacts of pathology, made this course feel like an actual medical school course. It was definitely a surreal moment.” (Unit 4 reflection)
Reflections on donor pathology and lifestyle	Exploring and visualizing diseases promoted reflection on one's own health and lifestyle choices	“After observing the various pathologies of our donors organ systems, I had to take a step back and think about my own health and what the state of my organs will be like in a few decades. These pathological findings have also reminded me that we only get one body, so we should take care of it.” (Survey)
General feedback on histopathology integration	Students recognized gaps in histology knowledge, wanted to collect more samples and spend more time reviewing slides	“Analyzing the histopathology with Dr. ___ was a great experience for connecting cellular mechanisms to pathology. But I wish we had more time discussing histology slides and taking different samples.” (Unit 2 reflection)

Students described how engaging with pathology and histopathology deepened their understanding of anatomy and anatomical relationships, particularly when visualizing pathology in one organ leading to comorbid diseases in other related organs. While most of the students were applying to or had been accepted to medical school, some described how engaging with histopathology opened the possibility of exploring some specialties (e.g., pathology and oncology) and confirmed interest in others. Students also described how they felt as if they were already in a medical school class because exploring pathology with whole body donors was not something they thought they would be able to do as undergraduate students.

Most of the comments relating to the impacts of the pathology thread described how physical engagement and exploration of pathology not only led to both reflections on the donor's health and how their pathology impacted their lifestyle, but also students reflected on how their own lifestyle choices may contribute to the diseases seen in their donors. While comments about histopathology were positive overall, students provided some suggestions for improving the thread through the collection of additional samples and being able to spend more time reviewing slides with the pathology content instructor.

## DISCUSSION

This study demonstrates an effective approach to integrating histopathology into an undergraduate gross anatomy curriculum and the impacts this curriculum integration has on the professional development of students. These results support others that have described the success of pathology and anatomy integration in medical education^5^, ^10^, ^11^ and its ability to promote clinical correlates between anatomy and pathology,^8^, ^9^ perceived improvement in student understanding of pathology,^21^ and increasing interest in medical specialties, such as pathology and laboratory medicine.^8^, ^22^ Furthermore, our experience with the quality of the specimens supports prior research that demonstrated embalmed donor tissues from the gross anatomy lab to be sufficient for educational and research purposes^23^ and that there are ample pathological tissues on whole body donors to support integration of histopathology in gross anatomy.^24^, ^25^, ^26^ Finally, considering the constraints of contact hours in professional curricula that can limit time for formal education on NTDIS, advanced, dissection‐based undergraduate gross anatomy courses may be a viable opportunity for the formal introduction of humanism through the reflection on the donor dissection experience and through exploration of pathology.

Hallam^27^ (p. 100) described “anatomising of human bodies after death,” in the context of medical education as a “relational process.” They further state, “Anonymization did not sever previous social relations… it remained latent, so the bodily scars and marks, for example, were seen by staff and students as indicators of the deceased's former life and relationships” (Hallam, 2017, p. 111). Analogous to our curricular intervention, the pathology and accompanying histopathology, arguably, enabled the latent pathologic processes that occurred in life to be explored supplying further indicators of the donor's life. Furthermore, this nuanced appreciation of the cellular changes of disease provided a path for students to move toward a new ethos of anatomy education, which “conceptualizes anatomy education as the first clinical experience”.^28^

### Lessons learned

During the implementation of the histopathology thread of the FPP, we learned several important lessons about implementing the project. The following sections are provided to furnish those who may wish to implement a similar curricular component into a new or already existing anatomy course by providing some recommendations that may assist in more straightforward implementation. These lessons are by no means exhaustive but are ones we thought may be useful or insightful when developing similar curricular initiatives.

First, considering the project was implemented in a semester‐long course, we recommend using local histology processing resources as much as possible to reduce the amount of time between tissue collection and slide viewing. One source of criticism from students was the delay between specimen collection and slide review, and this primarily occurred at the level of image digitization. Therefore, even though students prefer virtual over optic microscopy^29^ and virtual slides facilitated easier faculty review of slides with students, it may be more expedient to use glass slides and optic microscopes, particularly if faculty do not have access to an image scanner or are relying on other departments or laboratories to scan their slides.

Second, a project of this nature requires the expertise of individual(s) trained in pathology to review slides with students. While individuals trained in pathology may not be available at all institutions and the overall number of practicing pathologists has decreased in the last decade,^30^ pathologists' assistants are increasingly being utilized to provide anatomic pathology services under the direction of a qualified pathologist.^31^ Furthermore, digitization of slides affords the ability to review slides from almost any location.^32^ With the intended goal not to render a complete and thorough pathologic diagnosis, but instead to inform students understanding of cellular change associated with pathologic processes (i.e., inflammation, malignancy, and necrosis), any faculty versed in both normal histology and histopathology should be able to assist students in slide interpretation. Further, providing a list or guide of some of the more commonly encountered pathologies, such as emphysema, coronary atherosclerosis, lung neoplasm, liver cirrhosis, and benign prostatic hyperplasia to narrow students focus may be beneficial (see Geldenhuys et al., 2016 for a more detailed listing found in their study).

Third, the process of whole slide imaging and the subsequent digitization of slides for student viewing is costly.^32^ Indiana University School of Medicine is fortunate enough to have institutional research cores that are equipped with the resources and technology to prepare specimens and digitize histological images, so there were no additional costs associated with the purchase of equipment for this project. However, without grant funding, these services would be inaccessible to us. For future iterations, costs for slide processing could be distributed in the laboratory fees for students enrolled in the course. To reduce costs moving forward, a faculty member or graduate student could be trained to utilize the equipment to process the tissues and mount them onto the glass slides. We are exploring additional methods to improve the sustainability of this curricular intervention.

Fourth, given the number of slides for review per group (3–7) for each session, time became a barrier for sufficient discussion. While the students had taken a prerequisite anatomy course that included basic histology, students still only had a rudimentary appreciation for normal histology. As such, much of the time allocated with students was used to describe normal histology for the given organ and system, and the little remaining time was dedicated to basic histopathology. This initial iteration had the slide review occurring as needed, with dissection groups rotating every 10–15 min during laboratory dissections to discuss histopathology, with no formal time built into the schedule. For future iterations, having time set aside each week or every other week as dedicated histology slide review, aside from dissection, will allow for a more robust discussion and for students to become more well prepared. It is estimated that having 15–30 min built into their dissection schedule would be sufficient to meet this need.

Finally, as mentioned above, gaps in basic histology tissue morphology identification precluded a robust and in‐depth discussion of the encountered pathologies. To remedy this, optional, asynchronous histology modules on the four basic tissue types along with major systems would hopefully facilitate student learning of at least the basics of normal tissue morphology, if needed. Additionally, having an asynchronous general pathology module to introduce concepts of inflammation, necrosis, and classic hallmarks of malignancy would add a deeper understanding of the histopathology obtained from the students' anatomy donors.

### Limitations

This project was implemented with a single cohort of students at one institution, limiting the study's generalizability. Furthermore, the study is also limited by a small number of students completing the post‐course survey, which may have resulted in a positive response bias. Reflections contribute to a small percentage of the student grade (4%), and therefore, the reflections may have social desirability bias, where students could be sharing overly positive views of the FPP to appease instructors. Despite these limitations, this study presents an innovative approach to integrating histopathology into an undergraduate anatomy course, and our lessons learned may assist faculty in implementing a similar program at their institution.

## CONCLUSIONS

This curriculum enhancement initiative examined the role of histopathology in the FPP design and how it impacts undergraduate students' anatomic knowledge and professional development. Student reflections demonstrated that the histopathology component of the FPP improved clinical understanding of pathology, helped facilitate feelings of belonging in the medical profession, and allowed them to reflect on their own humanity as well as that of the donors. Several things were learned in the development of this curricular intervention that should be taken into consideration when moving forward. Time is required for the processing of tissues and digitization of slides, having a trained pathologist to collaborate with the students, and ensuring students understanding of basic tissue morphology. Additionally, resources may be limited as these services may be costly and require trained professionals. Using local histology resources can reduce the time and cost associated with tissue processing and digitization. Finally, setting aside dedicated time with a pathologist to explore pathology with students is likely to improve their experience. Despite barriers discovered in this initial iteration, the results support the integration of histopathology in undergraduate gross anatomy education.

## AUTHOR CONTRIBUTIONS

**Kyle A. Robertson:** Conceptualization; investigation; writing – original draft; methodology; writing – review and editing; data curation; supervision; project administration; funding acquisition; visualization. **Megan Kruskie:** Writing – original draft; writing – review and editing; project administration; investigation; data curation. **Jessica N. Byram:** Formal analysis; conceptualization; investigation; writing – original draft; writing – review and editing; methodology; project administration; supervision; data curation; funding acquisition.

## ETHICS STATEMENT

The authors have no conflicts of interest to disclose. This study received exempt status from Indiana University Institutional Review Board (IRB # 10962). This project was funded through a 2023 Curriculum Enhancement Grant from the Center for Teaching and Learning, Indiana University Indianapolis.

## Supporting information


Data S1.
