# Supplementary material for: Diagnosing the first patient: Integrating histopathology into an undergraduate gross anatomy course
Source: Anat Sci Educ. 2025 May 13;18(6):596–603. doi: 10.1002/ase.70042 (PMC12135458; doi:10.1002/ase.70042)
Supplement: Supplementary file 1 — Data S1. [file ASE-18-596-s001.docx]

**Supplemental Material A:** Exemplar first patient report from BIOL-N 461, Spring 2024 Cohort.

**Unit 3 Patient Report**

**Evidence of lifestyle –** This donor had multiple indications of lifestyle in the lower extremities. Firstly, our donor had bilateral total knee replacements. This was obvious on dissections as the distal femur was entirely made of what is likely cobalt-chromium. In addition, the patellas had been replaced with an entirely new material, the proximal tibias were replaced, and the menisci with a majority of the knee ligaments were gone. This double replacement at 69 years of age could be due to our donor’s occupation. They were a shipping manager and likely spent a lot of time standing, carrying heavy objects, bending, etc… that would likely deteriorate the knee joint quicker. Also, there was plenty of scar tissue around the knee, which could be due to surgeries or chronic inflammation. Another indicator of lifestyle was the donor’s feet. Although feet have the thickest skin on the body, this donor had extensive connective tissue that made the dissection very difficult. We suspect that the nature of the donor’s job also played a role in this finding. Lastly, the donor’s calf muscles, specifically the gastrocnemius, had considerable muscular density and color. This also indicates that this person was likely very active on their feet, and did a lot of things that regularly required extensive use of the calves.

**Pathology –** There were little signs of evidence of any sort of disease in the perineum, pelvic region, and lower extremities. The donor had pustules on both feet, but no sample was collected. The bladder was hard to touch when dissecting, and a sample was collected for further examination. The team observed that the donor had a hysterectomy, and no scar tissues developed over time. In addition, the donor also had two knee replacements and scar tissues were present when dissecting the popliteal and knee region.

However, a few days ago, the team received pathological slides with explanations regarding the tumor from the left hilum, lymph node, and tumor of the left. The c-rings of the bronchus contained a mix of normal respiratory epithelium and cancerous tissue. The slide also presented squamous carcinoma, described as the full, uncontrolled growth of cells; it exhibited larger nuclei than normal. The lymph node slide likely has a different stage of cancer cells. In addition, it contains lots of lymphocytes, which appeared to be dark purple on the slide. The tumor from the lung tissue contains a random cluster of cells with a pinkish, stringy pattern (termed desmoplasia, a fibrotic response to cancerous cells). Based on this observation, the desmoplasia may have caused a dense buildup of fibrotic tissue around the cancer cells and could be a response to radiation; however, this is not 100% certain.

**Impacts of Pathology**

The impacts of pathology on the patient is very limited for this unit. While going through the dissection there were minimal signs of pathology. However, our patient did have an abnormally hard bladder. This could result in the patient having urinary issues. With a harden bladder the patient would have a difficult time urinating as the bladder is no longer able to contract and expand to its full potential. The patient also had a double knee replacement, this produced a vast amount of scar tissue. The buildup of scar tissue could have caused reduced range of motion and created pain in the knee area. There were pustules on both feet which could have caused discomfort in walking or standing.

**Anatomic Variants**

Multiple significant surgical variants were observed throughout the donor’s lower limb and pelvic region. One of the most important variants was from a hysterectomy, which removed the donor’s ovaries and uterus; therefore, significant reproductive structures were not observed in the donor. Knee replacements were also observed in both of the donor’s knees. The condyles of the femur and tibia were both replaced by titanium counterparts, while the menisci were replaced with a plastic spacer leaving behind no remnants of the ACL or PCL. Potentially due to the knee replacement, the plantaris muscle was observed to be abnormally smaller in size.
